# Use of Olaparib in the Management of Metastatic Parathyroid Carcinoma With *BRCA1* Mutation

**DOI:** 10.1210/jcemcr/luaf007

**Published:** 2025-02-17

**Authors:** David Woodfield, Trang Le, Grace Prince, Hyun Lee, Hetal Vachhani, Priyanka Majety

**Affiliations:** Department of Endocrinology, Diabetes, and Metabolism, Virginia Commonwealth University, Richmond, VA 23298, USA; Department of Endocrinology, Diabetes, and Metabolism, Virginia Commonwealth University, Richmond, VA 23298, USA; Department of Endocrinology, Diabetes, and Metabolism, Virginia Commonwealth University, Richmond, VA 23298, USA; Department of Hematology, Oncology, and Palliative Care, Virginia Commonwealth University, Richmond, VA 23298, USA; Department of Hematology, Oncology, and Palliative Care, Virginia Commonwealth University, Richmond, VA 23298, USA; Department of Endocrinology, Diabetes, and Metabolism, Virginia Commonwealth University, Richmond, VA 23298, USA

**Keywords:** parathyroid carcinoma, BRCA, PARP inhibitor

## Abstract

Parathyroid carcinoma (PC) is a rare cause of primary hyperparathyroidism with a highly variable clinical course. We report the case of a man with metastatic PC who presented with severe hypercalcemia, discovered incidentally after a fall. He underwent left upper parathyroidectomy with left thyroid lobectomy, and pathology confirmed PC. After a year of absence from follow-up, he developed recurrence with bilateral pulmonary metastases. Cinacalcet and denosumab were initiated due to persistent, severe hypercalcemia, followed by wedge resection and palliative radiotherapy of pulmonary metastases. Genetic analysis revealed no actionable pathogenic variants, but a *BRCA1* mutation classified as a variant of unknown significance (VUS) was identified. He was started on olaparib, a poly adenosine diphosphate-ribose polymerase (PARP) inhibitor, 3 years after initial diagnosis. Following this, his PTH level declined by approximately 40% within 7 months. Subsequently, his PTH levels began increasing despite continuation of olaparib and, after 20 months, rose to his original PTH level prior to the initiation of therapy. This is a unique case of a patient with metastatic PC who had a *BRCA1* VUS mutation, with initial partial reduction in PTH and calcium levels after PARP inhibitor treatment.

## Introduction

Parathyroid carcinoma (PC) is a rare malignancy seen in 0.5% to 5% of patients with primary hyperparathyroidism [1]. Typically sporadic in occurrence, PC may also rarely occur as a feature of genetic syndromes including hyperparathyroidism-jaw tumor syndrome and multiple endocrine neoplasia types 1 and 2a. The diagnosis of PC is most commonly made postoperatively after parathyroidectomy for primary hyperparathyroidism, based on histopathological findings [1]. In patients presenting with PC, as many as 10% to 30% will have metastatic disease at the time of initial diagnosis [2]. The prognosis for metastatic PC is generally poor, though survival data are limited by the rarity of the disease. In a retrospective study of 79 patients with metastatic PC, median survival after diagnosis was 36 months, although survival ranged between 1 and 252 months [3].

Complete surgical resection of the tumor is the only known curative treatment of localized PC. Intraoperative findings suspicious for PC should prompt en bloc resection to avoid capsule rupture and excision of surrounding tissue to the lower risk of local recurrence [4]. Medical management of PC is aimed at reducing the effects of elevated PTH and, in doing so, reducing hypercalcemia, which is the primary cause of morbidity and mortality. This includes IV fluids, bisphosphonates, diuretics, and calcimimetic agents [1]. There is no clear consensus regarding the use of radiotherapy in PC. There have been no randomized controlled trials evaluating adjuvant radiotherapy, and retrospective studies have not shown benefit in survival [1]. The use of chemotherapy or immunotherapy is not part of the standard treatment of PC, as these have not been shown to be effective in either local or metastatic PC [4].

The molecular pathogenesis of PC is complex, with genetic analyses painting a highly heterogenous picture. One of the more commonly identified genetic mutations is *CDC73*, a tumor suppressor gene. This can be seen both as a germline mutation and, less commonly, as a somatic mutation. As a germline mutation, *CDC73* is also associated with fibro-osseous jaw tumors and renal or uterine masses, collectively known as hyperparathyroidism-jaw tumor syndrome [5]. Another more frequently identified mutation is in the *TP53* gene, responsible for the well-known tumor suppressor protein p53 [6]. To date, neither of these mutations has targeted therapies available.

Some mutations identified in PC, however, do have targeted therapies available. For example, up to 20% of PC samples have mutations in the PI3K/Akt/mTOR signaling pathway including *TSC1*, *PTEN*, and *PIK3CA* [7]. This pathway has been targeted in a patient with a *TSC1* mutation who was treated with an mTOR inhibitor (everolimus) and vascular endothelial growth factor inhibitor (vandetanib), resulting in an improvement in hypercalcemia and lack of disease progression [6]. Beyond the mTOR pathway, case studies have shown that targeting mutated *FGFR1* and *RET* with lenvatinib has led to complete biochemical remission. Additionally, targeting mutated *BRCA2* with olaparib, a poly adenosine diphosphate-ribose polymerase (PARP) inhibitor, has resulted in 14 months of disease control [8]. *BRCA1* and *BRCA2* encode proteins necessary for homologous recombination (HR), a method of DNA repair, and mutations of these genes lead to the loss of HR. Inhibition of PARP, which facilitates DNA repair, results in increased double-strand DNA breaks that are unable to be repaired without HR, resulting in increased cell death in *BRCA*-mutated cancer cells [9]. Data from a small review of 11 patients with advanced PC suggested that as many as 54% of cases harbor potentially actionable mutations [6]. In absence of effective chemotherapy or immunotherapy regimens, evaluating the therapeutic utility of actionable mutations found in PC holds the key to developing effective systemic treatment strategies.

## Case Presentation

A 36-year-old male presented to the emergency department following a right metacarpal fracture in the setting of a fall down a flight of stairs. Incidental laboratory findings showed hypercalcemia at 13.1 mg/dL (3.27 mmol/L) (normal reference range 8.2-10.2 mg/dL; 2.05-2.54 mmol/L). Following open reduction and internal fixation of a metacarpal fracture, he was discharged but subsequently returned to the emergency department 2 weeks later with a persistent headache and concern for pupillary abnormalities. Neurological evaluation was unrevealing, but laboratory assessment was notable for the progression of hypercalcemia to 14.8 mg/dL (3.69 mmol/L).

On further assessment, he reported several weeks of bone pain, constipation, and increased urinary frequency, but denied symptoms including changes in weight and abdominal pain. The patient's medical history included hypertension for which he was on amlodipine, and depression, for which he had been recently started on escitalopram. Before this, he had sustained a right hand and right ankle fracture in the setting of significant traumatic injury. He had no history of nephrolithiasis and no family history of hypercalcemia, nephrolithiasis, or malignancy.

## Diagnostic Assessment

At initial hospitalization, serum calcium was 14.8 mg/dL (3.69 mmol/L), with PTH elevated at 620.9 pg/mL (65.8 pmol/L) (normal reference range 9.0-77.0 pg/mL; 1.0-8.2 pmol/L). Neck ultrasound revealed a 1.7 centimeter hypoechoic, hypervascular nodule with irregular margins, located within or adjacent to the left thyroid lobe.

He then underwent left upper parathyroidectomy with left hemithyroidectomy due to concern for invasion into the left thyroid lobe, along with autotransplantation of left lower parathyroid gland to the right sternocleidomastoid muscle. Intraoperatively, PTH levels decreased from 352 pg/mL (37.3 pmol/L) to 33 pg/mL (3.5 pmol/L). Pathology revealed PC with benign thyroid tissue. The diagnosis of PC was supported by several factors including a high mitotic rate, intralesional fibrosis, and probable vascular invasion. Immunohistochemistry and next-generation sequencing tests completed on this tissue sample showed microsatellite stability, *PD-L1* combined positive score <1%, *PMS2 G1552A*, and *BRCA1* C2869G mutations. The *BRCA1* mutation was reported as a variant of uncertain significance (VUS).

Initial follow-up 4 months later revealed resolution of hypercalcemia [calcium 9.8 mg/dL (2.45 mmol/L)]. PTH was mildly elevated at 84.6 pg/mL (9.0 pmol/L), although in the context of low 25-OH vitamin D [11.1 ng/mL (27.7 nmol/L)] (normal reference range 30.0-100.0 ng/mL; 74.9-249.6 nmol/L), for which cholecalciferol 2000 international units daily was initiated.

He was lost to follow-up for over 1 year; upon re-establishing care, he was noted to have recurrent hypercalcemia [calcium 13.8 mg/dL (3.44 mmol/L)] and elevated PTH of 496.1 pg/mL (52.6 pmol/L). At that time, cinacalcet and denosumab were started for management of hypercalcemia. He had subsequent imaging, including a computed tomography chest, revealing multiple pulmonary nodules consistent with metastatic disease. He underwent wedge resection of a right lower lobe lung lesion, which confirmed metastatic PC. He also underwent stereotactic body radiation therapy to all remaining metastases in the left upper lung over the following 9 months.

## Treatment

At 3 years after initial diagnosis, the patient was seen in follow-up by oncology. He remained on cinacalcet and denosumab in addition to IV fluid infusions 3 to 4 times a week. In light of data in the field of breast cancer, which suggested that some VUS mutations of *BRCA1* respond at the same rates as pathologic mutations to PARP inhibitors, he was started on olaparib 300 mg twice daily.

## Outcome and Follow-up

**Figure 1.**
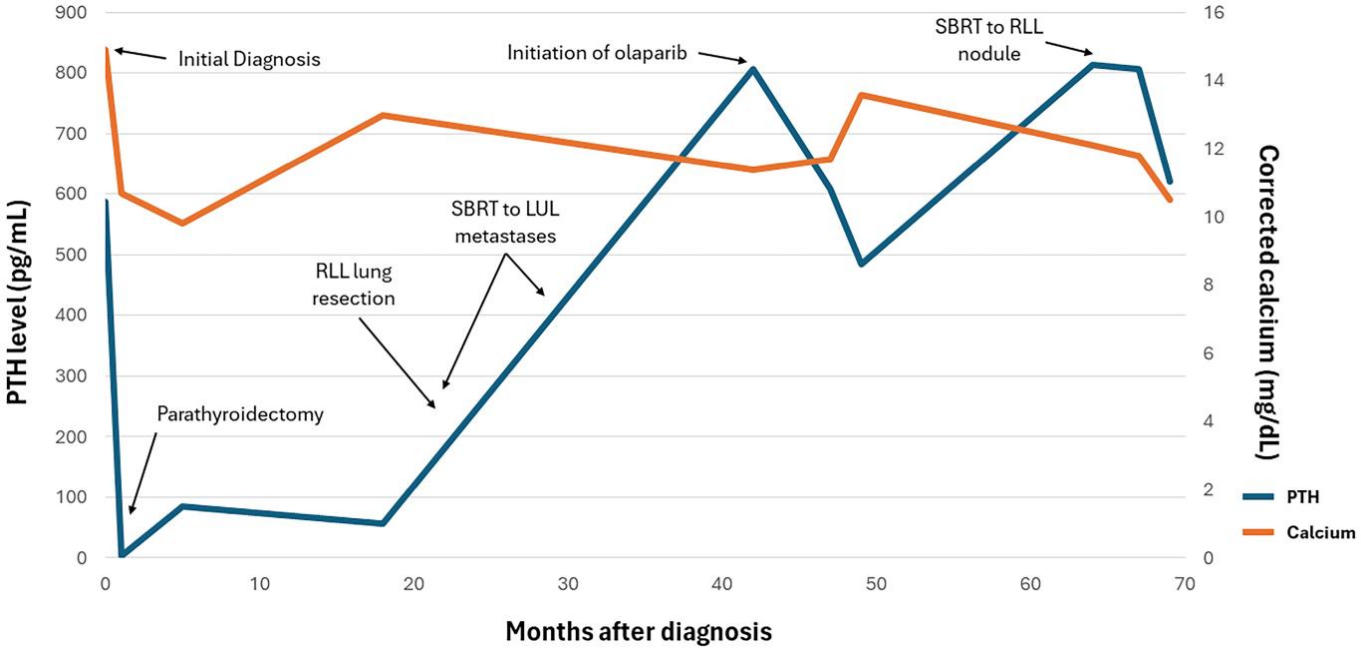
Timeline of PTH and albumin-corrected calcium in relation to diagnosis, procedural interventions, and olaparib therapy. Abbreviations: LUL, left upper lobe; RLL, right lower lobe; SBRT, stereotactic body radiation therapy.

Following initiation of olaparib, PTH levels declined from 806.4 pg/mL (85.5 pmol/L) to a nadir of 484.0 pg/mL (51.3 pmol/L) within 7 months (Fig. 1). He continued receiving IV fluid infusions at the same frequency, as well as cinacalcet and denosumab at the same doses. Despite this initial response, his PTH began to climb subsequently and 20 months after initiation of olaparib had risen to 814.3 pg/mL (86.4 pmol/mL). Follow-up whole-body computed tomography scans revealed an enlarging right lower lobe lung nodule measuring 18 × 15 mm. He underwent stereotactic body radiation therapy of this nodule with a subsequent decline in PTH, 2 months after treatment, to 621.8 pg/mL (65.9 pmol/mL).

## Discussion

Metastatic PC is an aggressive disease with limited established treatment options, currently focused on palliative treatment of the associated comorbidities related to hypercalcemia. We present a case of a patient with metastatic PC who was treated with a PARP inhibitor olaparib after being found to have a VUS mutation of *BRCA1*. There are very limited data in the literature regarding *BRCA* mutations in PC. Another case has been reported of a patient with PC who was found to have a pathogenic, somatic *BRCA2* mutation and was treated with PARP inhibitor olaparib with sustained partial response at 20 months [10]. In the case of the patient we reported, the *BRCA* mutation noted was *BRCA1* and was a VUS, rather than pathogenic. A retrospective study in 2019 showed no significant difference in progression-free survival between patients treated with olaparib in the setting of *BRCA* mutations that were either known to be pathogenic vs those that were classified as VUS [11].

This patient had an initial partial response with an approximately 40% decline in PTH levels following initiation of olaparib. The response was sustained for about 20 months before PTH returned to pretreatment levels, at which time a newly enlarging lung nodule was detected. This case adds to the body of literature that underscores the importance of investigating targeted therapies based on genetic mutations in the treatment of metastatic PC.

## Learning Points

- Genetic testing in rare malignancies such as PC may reveal actionable targets, providing the potential for additional therapeutic options.
- PARP inhibitors, such as olaparib, are a reasonable systemic treatment option to consider for metastatic PC with BRCA1 mutation.
- Patients with VUS mutation of BRCA1 may still benefit from therapies targeting defective homologous recombination DNA repair.

## Data Availability

Original data generated and analyzed during this study are included in this published article.
